# Supplementary material for: Mesoscale computational protocols for the design of highly cooperative bivalent macromolecules
Source: Sci Rep. 2020 May 14;10:7992. doi: 10.1038/s41598-020-64646-5 (PMC7224399; doi:10.1038/s41598-020-64646-5)
Supplement: Supplementary file 1 — Supplementary information. [file 41598_2020_64646_MOESM1_ESM.pdf]

Mesoscale computational protocols for the design of highly cooperative bivalent macromolecules *Supplemematary Information*

Suman Saurabh[1,*] **and Francesco Piazza**[1,2]

[1]Centre de Biophysique Moléculaire, CNRS, Rue Charles Sadron, 45071, Orléans, France.
[2]Université d'Orléans, Pôle de Physique, 45071, Orléans, France.
[*]suman.saurabh@cnrs-orleans.fr

The angle-bending coefficient for the coarse-grained model of the linker

The bond-bond correlation function (BBCF) for a freely rotating chain is an exponentially decaying function of the monomer-monomer separation along the chain[1],

$$\langle \vec{t}_{i+m} \cdot \vec{t}_i \rangle = a^2 \left(\cos\theta\right)^m = a^2 e^{-ma/\ell_p} \tag{1}$$

where ℓ_p is the chain persistence length,

$$\ell_p = -\frac{a}{\log(\cos\theta)} \tag{2}$$

In Eq. (1), the average is taken over the free rotations about the segment axes (free torsions) and θ is the angle formed by two successive segments, $\cos\theta = a^{-2}\vec{t}_{i+1}\cdot\vec{t}_i$, $|\vec{t}_i| = a$ (see Fig. S1).

If the angle θ is no longer fixed but is controlled by a potential energy $V(\theta)$, in the hypothesis that free torsions along ϕ and bending are uncoupled degrees of freedom along the chain, the BBCF is still an exponentially decaying function and the persistence length can be computed as

$$\ell_p = -\frac{a}{\log\langle\cos\theta\rangle} \tag{3}$$

where

$$\langle\cos\theta\rangle = \frac{\int_0^\pi \cos\theta \sin\theta \, e^{-V(\theta)/k_BT}}{\int_0^\pi \sin\theta \, e^{-V(\theta)/k_BT}} \tag{4}$$

The calculation can be carried out explicitly for example in the case of the cosine angle potential, $V(\theta) = k_\theta(1-\cos\theta)$, which reduces to an harmonic bending potential for a semiflexible polymer. From eq. (4), one gets

$$\langle\cos\theta\rangle = \frac{\alpha - 1 + (\alpha+1)e^{-2\alpha}}{\alpha(1-e^{-2\alpha})} \tag{5}$$

where $\alpha = k_\theta/k_BT$. For PEG the persistence length is $\ell_p = 3.8$ Å while the monomer length is $a = 1.5$ Å[2]. In our coarse-grained representation the monomer size is 3.5 Å, which would set a lower bound on the angle bending energy $k_\theta \simeq k_BT$. In our simulations we fixed $k_\theta = 1.8\,k_BT$, which we used as the bending coefficient for the linker (see Fig. S1, right panel).

References

1. See for example M. Rubinstein and R. H. Colby, Polymer physics, Oxford University Press (2003).

2. H Lee, R M Venable, A D MacKerell Jr., and R W Pastor, Biophys J. **95**(4), 1590−1599 (2008).

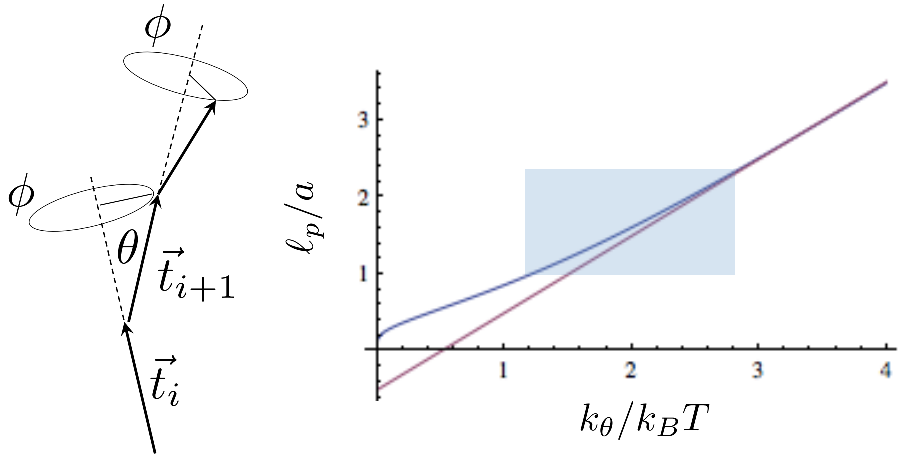

Figure S1. Left: section of a freely rotating chain. Right: plot of the function (3) with Eq. (5) (blue). The purple line is a plot of the stiff polymer approximation $\ell_p = a(k_\theta/k_BT - 1/2)$. The transparent rectangle identifies the region $\ell_p/a \in [1, 2.5]$ and the corresponding values of $\alpha = k_\theta/k_BT$.

Table S1. Flight/Residence times SPH system, repulsive wall, s_{wall} for nbd-2 = 4.5

Linker length	Flight time (ns)	Residence time (ns)
10	69.50 ± 1.92	3.24 ± 0.04
20	68.88 ± 1.10	3.37 ± 0.08
30	79.18 ± 3.05	3.39 ± 0.04
40	88.26 ± 2.22	3.45 ± 0.04
50	89.38 ± 6.45	3.42 ± 0.05
60	97.45 ± 6.90	3.47 ± 0.05

Table S2. Flight/Residence times SPH system, repulsive wall, s_{wall} for nbd-2 = 3.0

Linker length	Flight time (ns)	Residence time (ns)
10	59.90 ± 0.67	2.93 ± 0.07
20	62.96 ± 0.75	3.21 ± 0.02
30	73.83 ± 2.50	3.34 ± 0.04
40	82.66 ± 3.38	3.44 ± 0.04
50	88.20 ± 6.12	3.38 ± 0.08
60	94.89 ± 5.30	3.49 ± 0.04

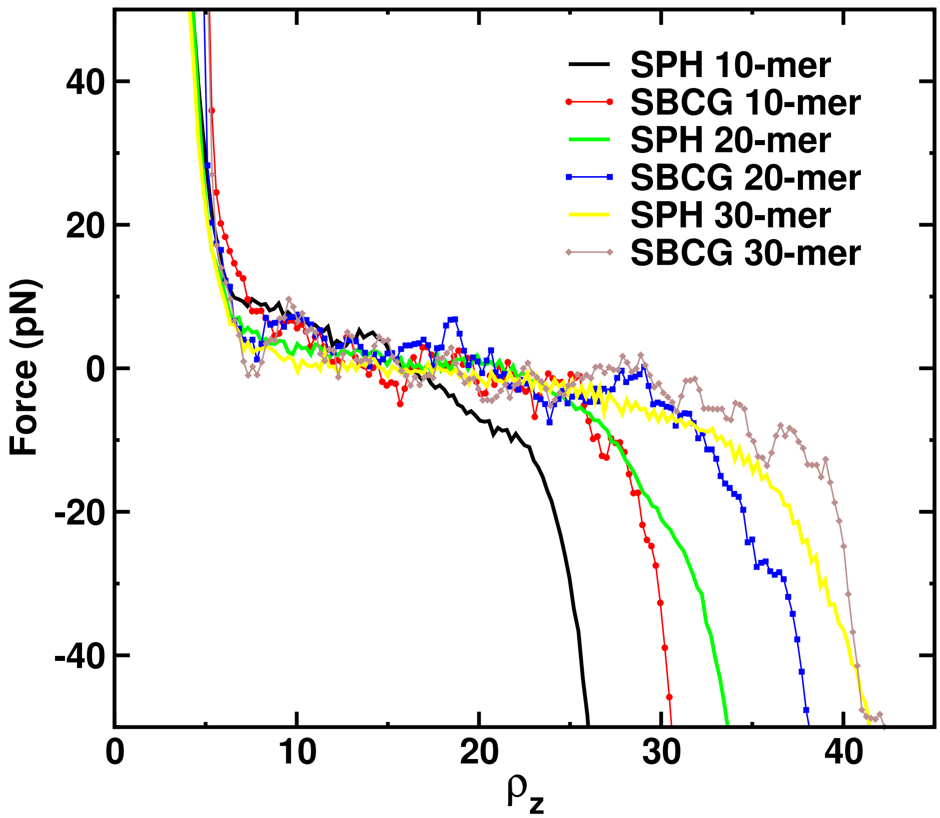

Figure S2. **Force**: Derivative of the PMF profiles as a function of ρ_z leading to the force on nbd-1 for a given value of the RC. The SBCG system experiences larger forces near the wall.

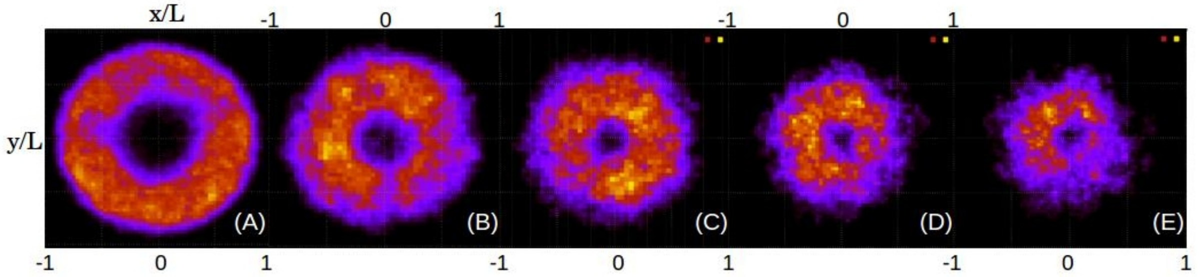

Figure S3. The xy distribution of the paratope position averaged over 25 diabodies with the condition that the paratope be within a distance of 5 σ from the lower wall of the box for linker lengths of (A) 10, (B) 20, (C) 30, (D) 40 and (E) 50 monomers.

Table S3. **Flight/Residence times** SPH system, attractive wall, $\varepsilon = 1.5k_BT$, s_{wall} for nbd-2 = 4.5

Linker length	Flight time (ns)	Residence time (ns)
10	42.00 ± 1.53	4.76 ± 0.06
20	37.20 ± 1.67	4.76 ± 0.03
30	45.50 ± 1.61	4.83 ± 0.05
40	48.31 ± 1.24	4.81 ± 0.06
50	51.15 ± 0.92	4.82 ± 0.06
60	53.84 ± 5.89	4.85 ± 0.04

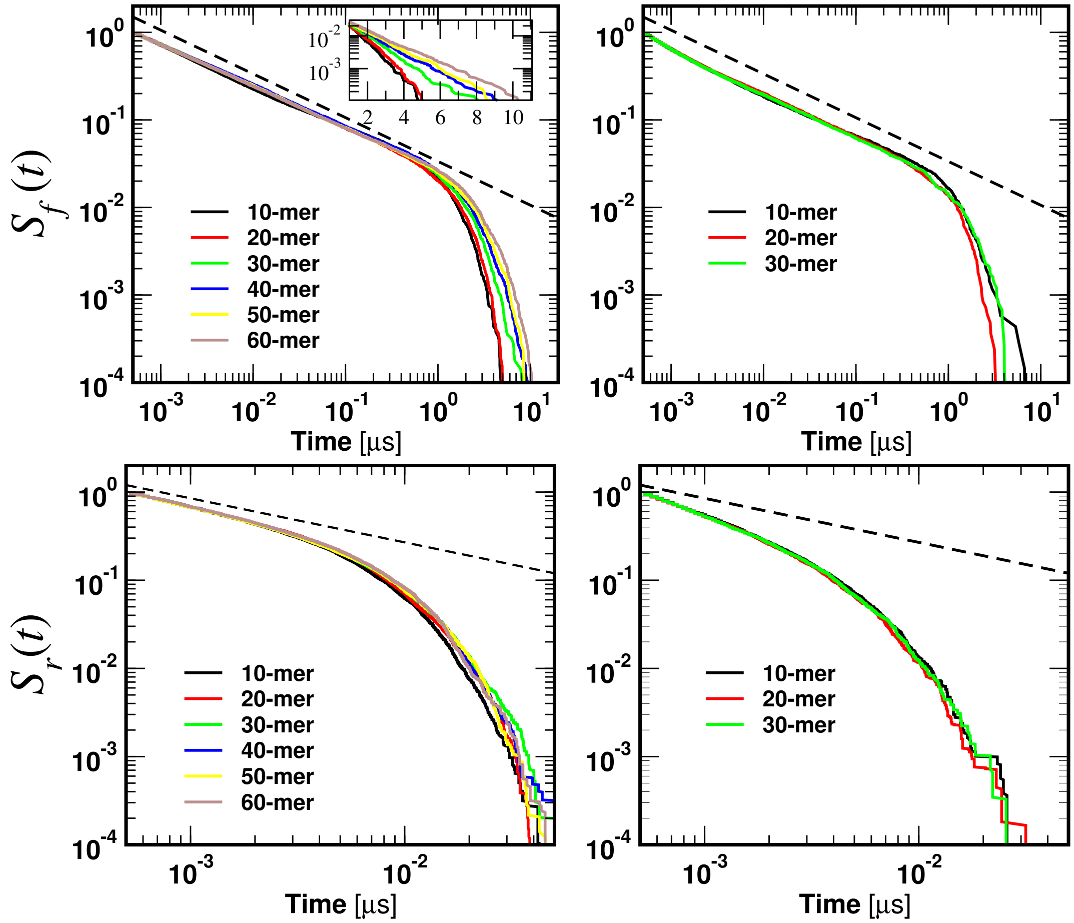

Figure S4. (Color online) **Survival probabilities.** Survival probability of the paratope in the flight ($z \geq z_{th}$, top) and residence ($z < z_{th}$, bottom) domains for (left) the SPH system and (right) SBCG system for different linker lengths. The dashed lines are plots of power laws of the kind $t^{-1/2}$. The inset shows a close-up in lin-log scale of the tails of the flight survival probability, which makes the exponential decay clearly visible. The plots for the attractive walls are provided in Fig. S5.

Table S4. Flight/Residence times SPH system, attractive wall, $\varepsilon = 2.5k_BT$, s_{wall} for nbd-2 = 4.5

Linker length	Flight time (ns)	Residence time (ns)
10	26.27 ± 0.70	7.52 ± 0.06
20	23.27 ± 0.45	7.10 ± 0.06
30	27.89 ± 1.39	7.10 ± 0.11
40	29.54 ± 1.56	6.90 ± 0.03
50	32.71 ± 1.67	7.14 ± 0.14
60	32.06 ± 2.50	7.30 ± 0.13

Table S5. Flight/Residence times SBCG system, repulsive wall

Linker length	Flight time (ns)	Residence time (ns)
10	53.72	1.90
20	45.03	1.83
30	49.50	1.86

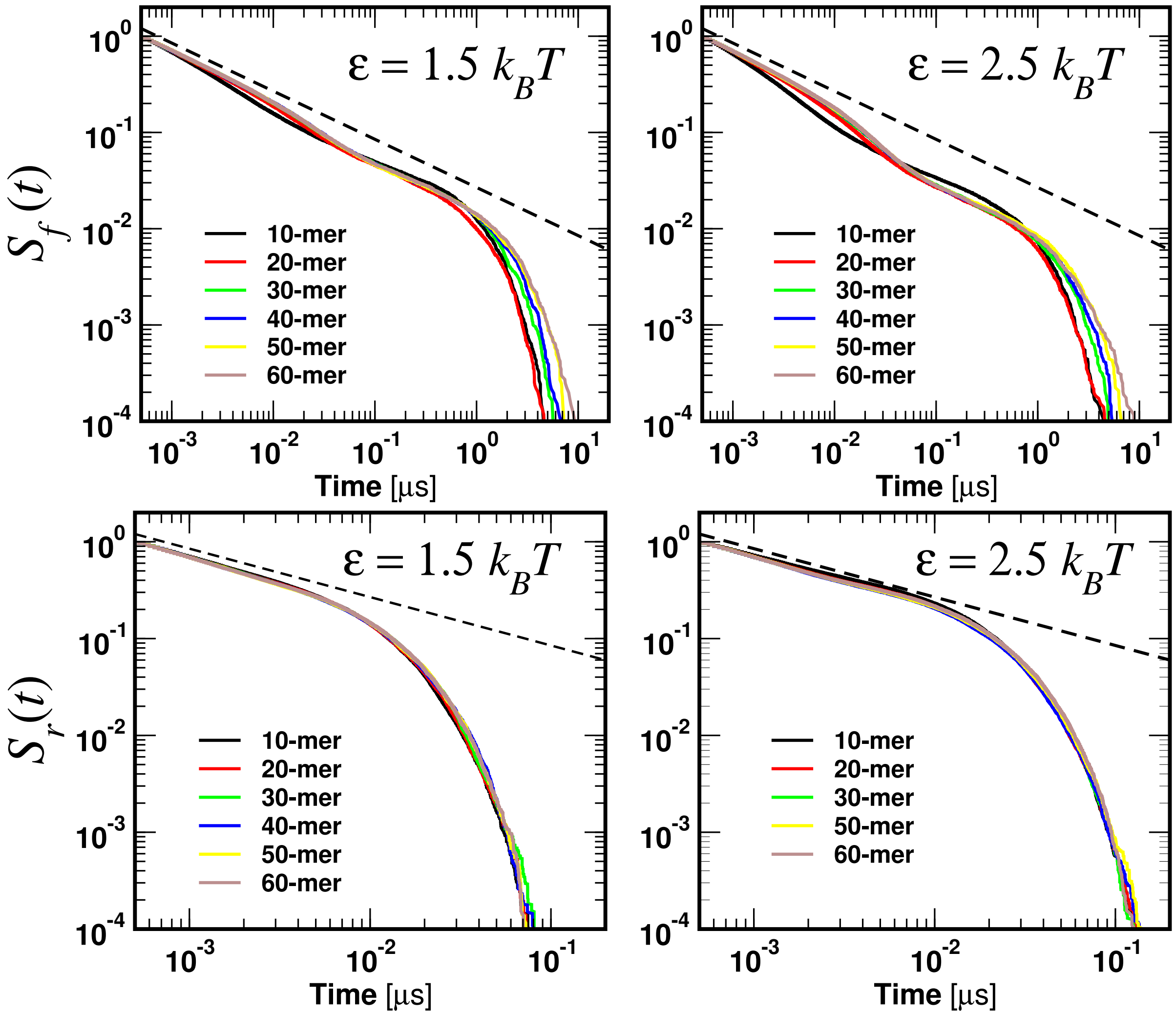

Figure S5. (Upper panel) Survival probability of the epitope in the flight ($z \geq z_{th}$) domain for the SPH (left) system and (right) SBCG system for different linker lengths. (Lower panel) Survival probability of the epitope in the residence ($z < z_{th}$) domain for the SPH (left) system and (right) SBCG system for different linker lengths. The dashed lines are plots of a power law of the kind $t^{-1/2}$.

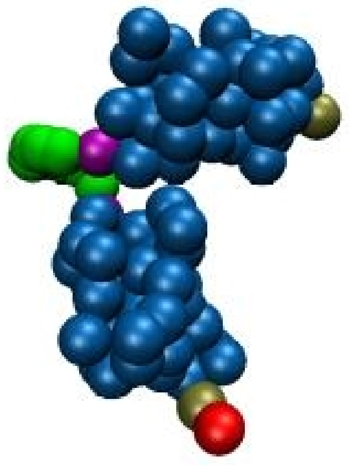

Figure S6. Steric repulsion between nbd-1 and nbd-2 for the 10-mer linker preventing nbd-1 from reaching close to the tethering wall.

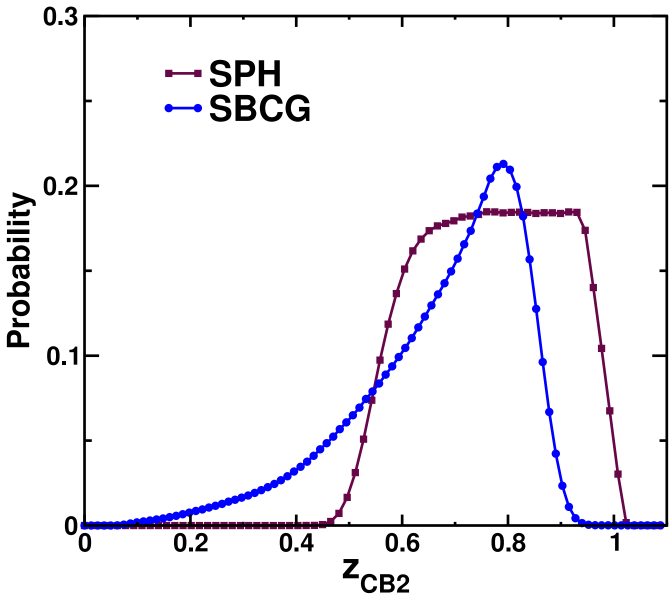

Figure S7. Distribution of the normalized height of CB2 from the tethering wall for the 10-mer SPH system (repulsive wall, s_{wall} = 4.5 for nbd-2) and the 10-mer SBCG system.

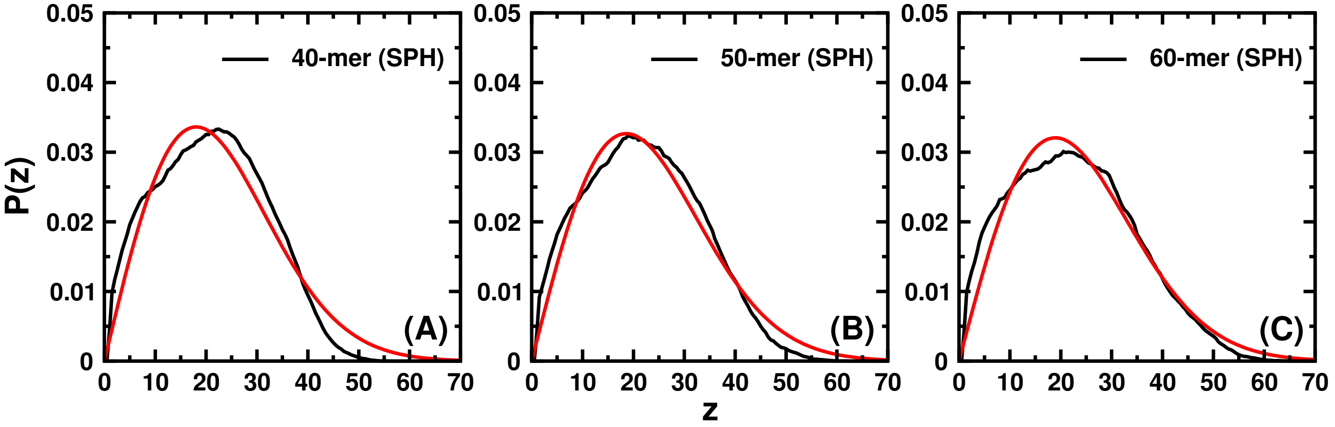

Figure S8. Distribution of the z-coordinate of P1 for (A) 40-, (B) 50- and (C) 60-mer systems along with the fits made using Eq. (8) in the main text with N_k as the free parameter.

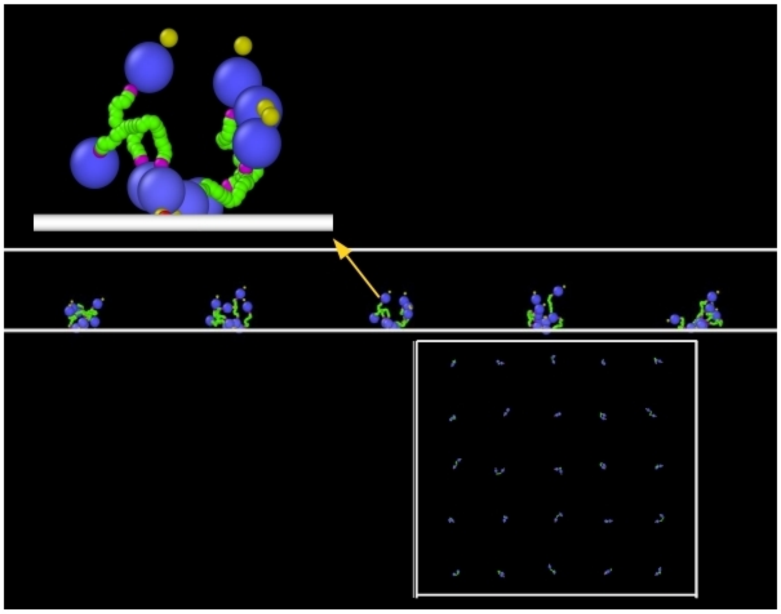

Figure S9. **Simulation for flight/residence time calculation:** Tethered 20-mer SPH diabodies in a 5×5 lattice. A similar lattice arrangement was used for all other systems (SBCG systems and SBCG systems with all other linker lengths). The distance between neighboring diabodies was increased as the linker length increased to avoid any interaction between neighbors.

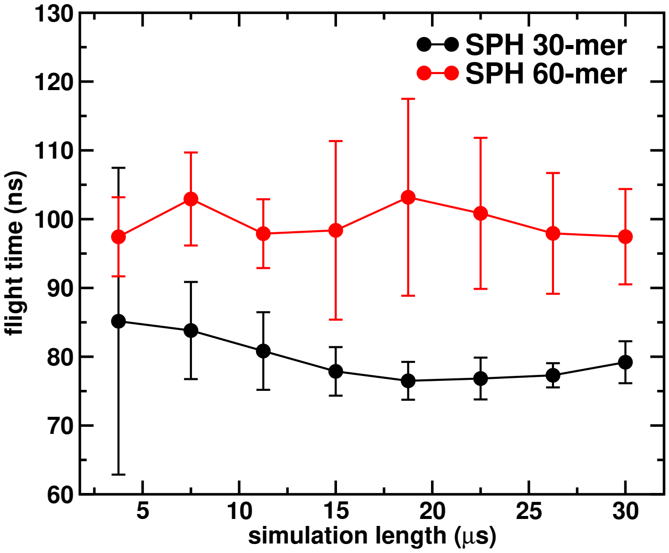

Figure S10. Flight time as a function of simulation length.
